# Gallstone Ileus Occurring 35 Years After Cholecystectomy in an Elderly Male

**DOI:** 10.7759/cureus.96985

**Published:** 2025-11-16

**Authors:** Michael W Alchaer, Tyler Stevens, Yaima Valdes, Andres Vidovich Ortiz, Thomas A Abbruzzese

**Affiliations:** 1 General Surgery, HCA Healthcare/University of South Florida (USF) Morsani College of Medicine Graduate Medical Education (GME) HCA Florida Brandon Hospital, Brandon, USA

**Keywords:** biliary-enteric fistula, cholecystectomy, enterolithotomy, gallstone ileus, small bowel obstruction

## Abstract

Gallstone ileus occurring decades after cholecystectomy is exceptionally rare and represents an uncommon cause of small bowel obstruction (SBO) in elderly patients with prior gallstone disease.

A 72-year-old male with a remote history of cholecystectomy presented with nausea, vomiting, and diffuse abdominal pain. CT imaging demonstrated an SBO with a calcified intraluminal mass in the distal ileum. After initial conservative management, the patient underwent exploratory laparotomy with extensive adhesiolysis, enterolithotomy, and primary ventral hernia repair. A large, calcified gallstone was removed from the distal ileum, confirming gallstone ileus. Postoperatively, the patient developed a transient superficial wound infection managed with bedside debridement and antibiotics, with full recovery and discharge on postoperative day ten.

Gallstone ileus many years following cholecystectomy likely results from a persistent, chronically developed biliary-enteric fistula enabling delayed stone passage. Its nonspecific presentation and rarity can delay diagnosis, highlighting the importance of maintaining a high index of suspicion in elderly patients presenting with SBO and intraluminal calcified densities.

This case emphasizes that gallstone ileus can occur long after cholecystectomy. Early recognition and timely operative intervention remain essential for optimal outcomes.

## Introduction

Gallstone ileus is a rare but clinically significant cause of small bowel obstruction (SBO), representing 1-4% of all SBO cases and up to 25% among elderly patients [1,2]. It typically results from a cholecystoenteric fistula that allows gallstones to migrate into the intestinal lumen, most often lodging within the terminal ileum [3]. Although gallstone ileus primarily occurs in patients with an intact gallbladder, its development decades after cholecystectomy has been reported only rarely [4-8]. Proposed mechanisms include persistent or recurrent biliary-enteric fistulas, retained or spilled gallstones, and de novo enterolith formation within the bowel lumen [9-13]. Because of its rarity and nonspecific presentation, diagnosis is often delayed, particularly in elderly patients with a history of biliary surgery [14]. This case illustrates gallstone ileus presenting 35 years after cholecystectomy, emphasizing the importance of maintaining a high index of suspicion in similar patients [15].

## Case presentation

A 72-year-old male with a history of diabetes mellitus, hypertension, coronary artery disease, and prior diverticulitis presented with a three-day history of nausea, vomiting, diffuse abdominal pain, and generalized weakness. His surgical history included a cholecystectomy performed in the 1980s, coronary artery bypass grafting, left below-knee amputation, right transmetatarsal amputation, and multiple vascular interventions.

On admission, the patient was hemodynamically stable. Abdominal examination revealed mild distension without signs of peritonitis. Laboratory evaluation demonstrated leukocytosis (white blood cell count 15.4 × 10³/µL), anemia (hemoglobin 10.3 g/dL), acute kidney injury (creatinine 6.9 mg/dL), and metabolic acidosis. Initial computed tomography (CT) imaging revealed dilated small bowel loops measuring 5.3 cm in diameter and an intraluminal hyperdense lesion. Conservative management with nasogastric decompression and intravenous fluids was initiated; however, the patient’s symptoms worsened, with persistent emesis.

**Figure 1:**
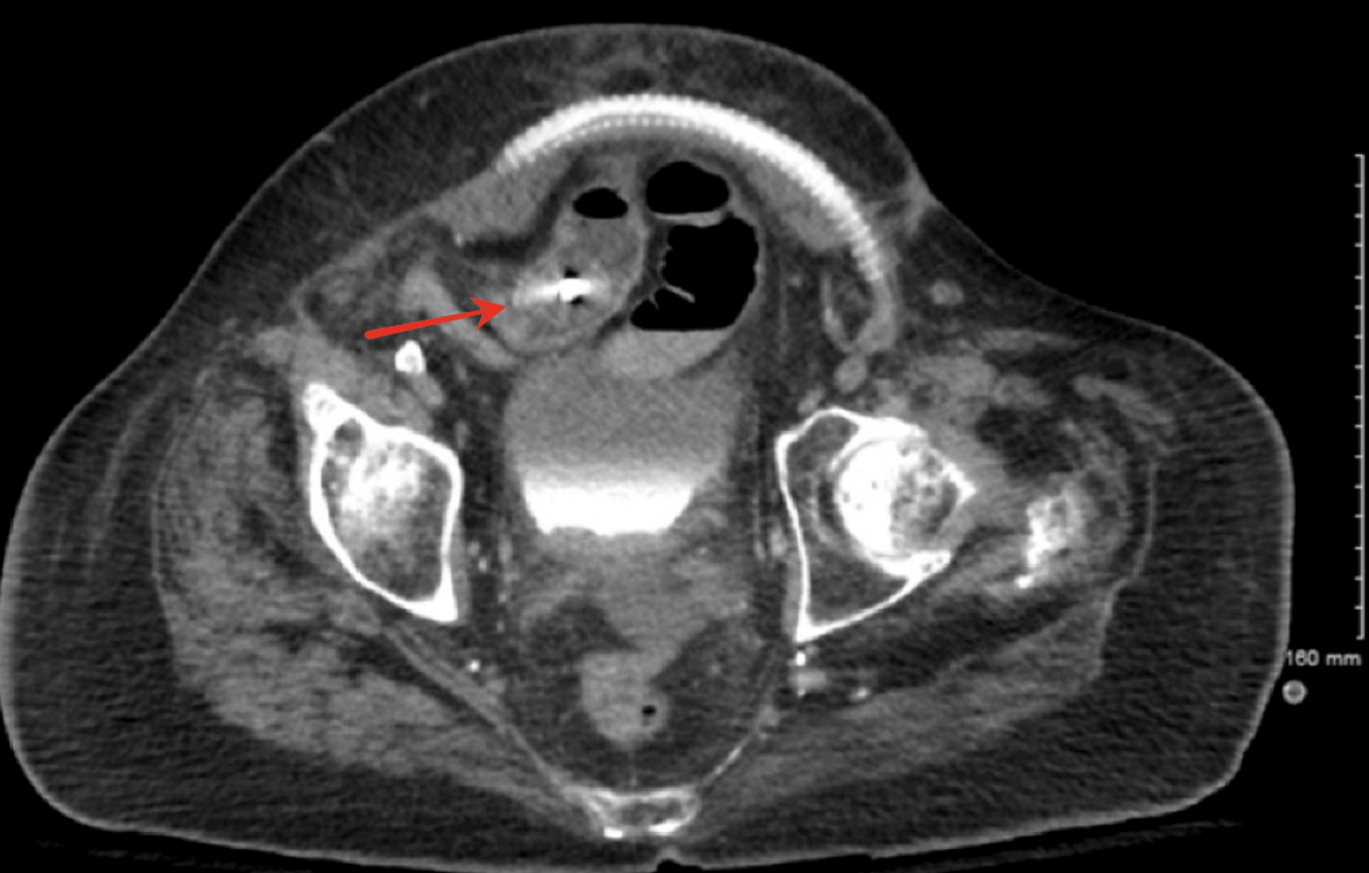
Axial CT image of the abdomen and pelvis with oral contrast demonstrating a calcified intraluminal gallstone causing small bowel obstruction (transition point indicated)

**Figure 2:**
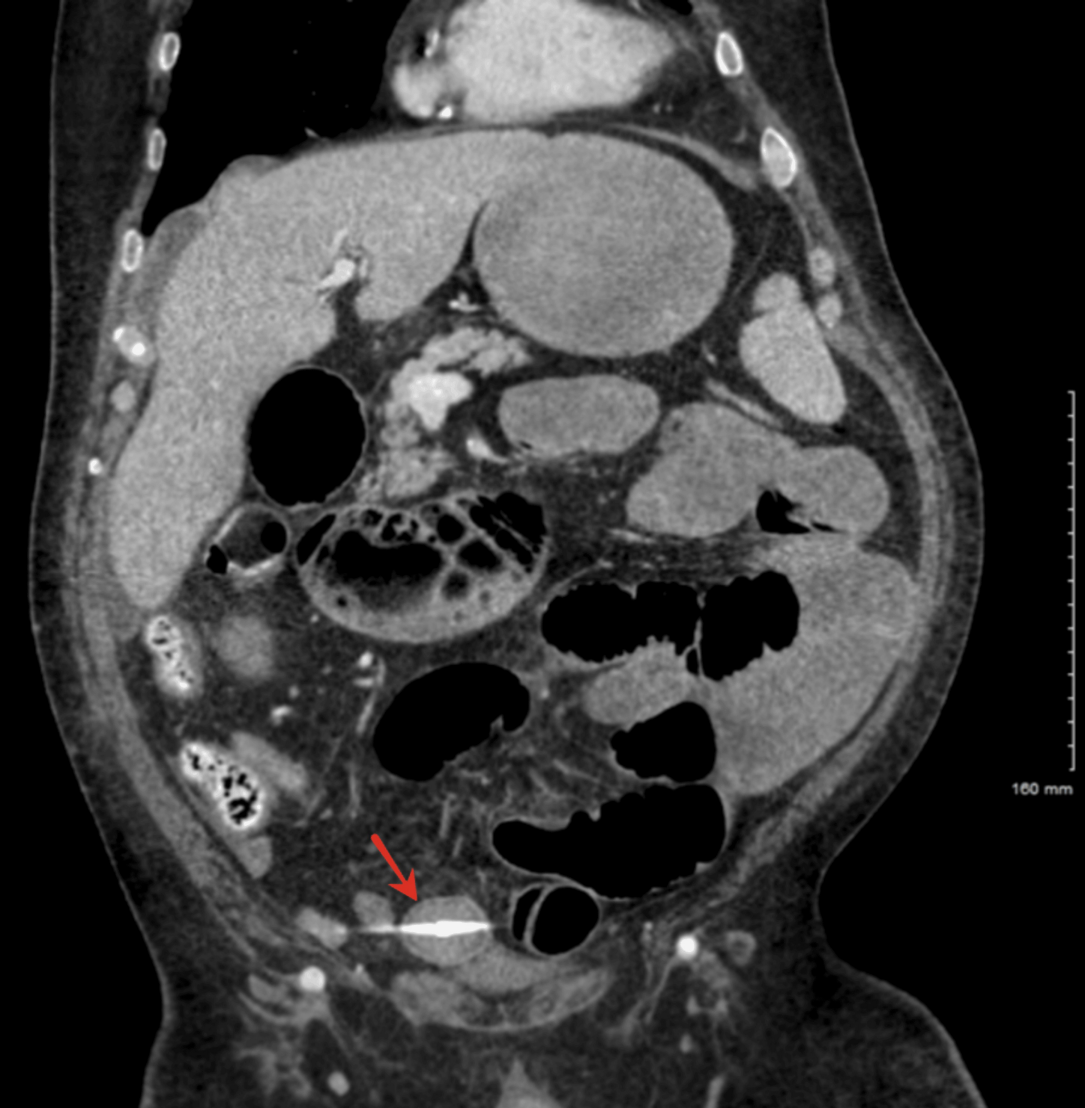
Coronal CT image of the abdomen and pelvis with oral contrast showing the obstructing gallstone within the distal ileum, consistent with gallstone ileus

A repeat CT with oral contrast demonstrated multiple colonic diverticula and a small bowel obstruction with a transition point in the lower central abdomen (Figure 1, Figure 2). The obstruction correlated with the level of a calcified intraluminal object in the distal ileum.

**Figure 3:**
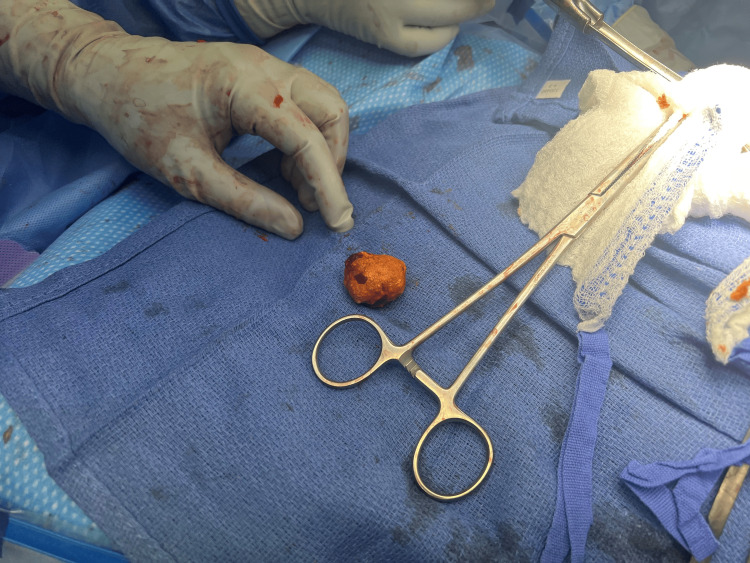
Operative photo of the extracted gallstone measuring 3.6 × 3.1 × 2.6 cm, demonstrating an irregular, calcified surface

After obtaining informed consent, the patient underwent exploratory laparotomy with extensive adhesiolysis lasting over two hours, enterolithotomy, and repair of multiple ventral hernias. Intraoperatively, a large, calcified gallstone was identified and removed via longitudinal enterotomy, which was then closed in two layers. A Jackson-Pratt drain was placed, and the specimen was sent for chemical analysis of stone. Stone analysis results revealed a green, irregular, firm cholesterol gallstone measuring 3.6 × 3.1 × 2.6 cm (Figure 3).

Postoperatively, the patient required prolonged nasogastric decompression due to high bilious output before bowel function resumed. The Jackson-Pratt drain was removed on postoperative day five. Discharge was delayed by a superficial wound infection requiring bedside debridement and intravenous antibiotics (vancomycin and piperacillin-tazobactam). The patient’s condition improved, and he was discharged home in stable condition on postoperative day 10.

## Discussion

Gallstone ileus is an uncommon cause of SBO, accounting for approximately 1-4% of all cases and up to 25% in elderly patients [1,2]. The condition typically develops when a large gallstone erodes through the gallbladder wall into the intestinal lumen, most often through a cholecystoenteric fistula [3]. Although this entity classically occurs in patients with an intact gallbladder, several reports have described its development years or even decades after cholecystectomy [4-8].

The pathophysiology of post-cholecystectomy gallstone ileus remains unclear. The most widely accepted explanation is the persistence or recurrence of a biliary-enteric fistula that allows late passage of a gallstone into the bowel [9-11]. Other mechanisms include retained or spilled gallstones during previous surgery or de novo enterolith formation in a chronically stagnant bowel segment [12,13]. Regardless of etiology, delayed migration of a large, calcified stone can ultimately cause distal ileal impaction and mechanical obstruction.

CT is the diagnostic modality of choice, as it can demonstrate Rigler’s triad - mechanical obstruction, pneumobilia, and an ectopic gallstone - with greater sensitivity than plain radiography or ultrasound [14,15]. CT also provides valuable preoperative information regarding the level of obstruction and associated complications such as ischemia or perforation [14,15]. Early recognition on CT significantly improves outcomes by expediting surgical management [16].

Surgical intervention remains the cornerstone of therapy for gallstone ileus [17]. Enterolithotomy alone is generally preferred in elderly or high-risk patients, as it provides rapid relief of obstruction with lower physiologic stress [18]. In stable patients, a one-stage procedure incorporating enterolithotomy, cholecystectomy, and fistula repair may be considered, though it carries a higher operative risk and longer duration [19,20].

This case demonstrates one of the longest latency periods reported for gallstone ileus after cholecystectomy. It underscores the importance of maintaining gallstone ileus in the differential diagnosis for SBO in elderly patients with a remote history of biliary surgery. Prompt diagnosis with CT imaging and timely surgical intervention remain essential to ensure favorable outcomes.

## Conclusions

Gallstone ileus remains a rare but important cause of small bowel obstruction, particularly in elderly patients. Although it most commonly occurs in those with an intact gallbladder, it can develop decades after cholecystectomy due to persistent or recurrent biliary-enteric communication. A high index of suspicion is essential when evaluating bowel obstruction in patients with a remote history of biliary surgery. Early recognition with computed tomography and timely surgical intervention are crucial to prevent complications and ensure optimal patient outcomes. In select cases, postoperative magnetic resonance imaging (MRI) can be considered to further evaluate potential underlying biliary-enteric communications or retained stones, thereby addressing the etiology and guiding long-term management.
